# Benign foveal retinal pigment epithelium hypopigmentation without functional loss : pediatric case series

**DOI:** 10.1007/s00417-025-07074-3

**Published:** 2026-01-10

**Authors:** Emilie Boulert, Isabelle Drumare, Claire-Marie Dhaenens, Sabine Defoort-Dhellemmes, Vasily Smirnov

**Affiliations:** 1 Service d’Exploration de la Vision et de Neuro-Ophtalmologie, CHU Lille, Lille, 59000 France; 2 Univ. Lille, CHU Lille, U1172-LilNCog-Lille Neuroscience & Cognition, Inserm, Lille, 59045 France

**Keywords:** Benign foveal depigmentation, Pediatric case-series, Multimodal retinal imaging, Functional assessment, Genetic testing

## Abstract

**Introduction:**

We report a case series of pediatric patients with incidentally discovered atypical maculopathy, namely, benign foveal depigmentation of the retinal pigment epithelium (BFD). Longitudinal data, both functional and morphological, were analyzed.

**Materials and methods:**

Functional tests (perimetry, full-field, multifocal and pattern ERG) were performed using the Mon PackOne® unit (Métrovision, Pérenchies, France), and multimodal retinal imaging was carried out using the Heidelberg Retinal Tomograph 2 and Heidelberg Spectralis SD-OCT (Heidelberg Inc., Germany).

**Results:**

Four patients were examined, two of whom were followed longitudinally. Pattern ERG, multifocal ERG, and central static perimetry were all within normal limits. The reflective structure of the retinal layers in the foveolar zone appeared normal on SD-OCT, except for a cone of choroidal hyperreflectivity corresponding to the lesion site. The lesion was more evident on near-infrared autofluorescence imaging, presenting as a well-defined, round, hypoautofluorescent foveal area. No progression was observed in the two patients for whom follow-up data were available.

**Conclusion:**

BFD is a rare and sporadic condition that may affect both adults and children. It has no functional visual impact. However, long-term follow-up is advisable to confirm the benign and non-progressive nature of these depigmented macular lesions.

**Supplementary Information:**

The online version contains supplementary material available at 10.1007/s00417-025-07074-3.

## Key messages

**What is known:**

- Few cases in the literature, mostly adults
- No functional loss
- Focal alteration of melanin synthesis/distribution
- Differential with Stargardt disease

**What is new:**

- Largest pediatric case series
- Multimodal imaging and functional assessment
- Longitudinal data showing no progression
- Genetic testing on large IRD panel negative

**Supplementary Information:**

The online version contains supplementary material available at 10.1007/s00417-025-07074-3.

## Introduction

Benign foveal depigmentation (BFD) is a rare and poorly understood retinal condition. It is characterized by the ophthalmoscopic appearance of maculopathy without any functional visual impairment. Only ten cases of BFD have been described to date in adults [1–3]. Pediatric cases are more rare, with only two patients reported [4]. Here, we present functional and multimodal imaging findings in a series of pediatric patients diagnosed with BFD.

## Patients and methods

This is a retrospective study based on data from patients followed in *Exploration de la Vision et Neuro-Ophtalmologie* Department, a national Reference Center for Rare Ocular Diseases at Lille University Hospital, Lille, France. Informed consent was obtained from all patients and their parents. The study was approved by the CPP Nord-Ouest ethics committee and complies with the principles of the Declaration of Helsinki.

The following data were collected: best-corrected visual acuity (BCVA), slit-lamp examination, and fundus. Static perimetry was performed using MonCV One^®^ system (Métrovision, Pérenchies, France). Full-field, multifocal, and pattern electroretinograms (ERGs) were conducted with MonPackOne^®^ system (Métrovision, Pérenchies, France) in accordance with ISCEV standards [5]. Multimodal imaging included fundus photography using the Clarus 500 (Zeiss, Oberkochen, Germany); infrared reflectance (IRR), short-wavelength autofluorescence (SWAF), and near-infrared autofluorescence (NIRAF) imaging were performed using the HRA2 system (Heidelberg Inc., Germany). Spectral-domain optical coherence tomography (SD-OCT) was performed using the Spectralis SD-OCT system (Heidelberg Inc., Dossenheim, Germany).

Two patients (P2 and P3) underwent genetic testing using a next-generation sequencing (NGS) panel targeting 230 genes associated with inherited retinal dystrophies (IRD) [6]. Capture oligonucleotide probes were designed using the Haloplex target enrichment System (Agilent Technologies Inc., Santa Clara, CA, USA). DNA libraries were sequenced on a NovaSeq sequencer (Illumina Inc., San Diego, CA, USA). The data analysis was conducted using an in-house developed pipeline compiling the data obtained from Seqnext (JSI Medical System, Ettenheim, Germany) and GATKsoftware.

## Results

Clinical findings are summarized in Table 1.

**Table 1:** Clinical findings of the patients included in case

	Age at first examination (y.o.)	BCVA, decimals	Anterior segment	Visual Field	mfERG	pERG	ffERG	Fundus	SWAF	NIRAF	SD-OCT	Follow up period (years)
P1	3	6/10	normal	-	-	-	normal	Small bilateral hypopigmented foveolar lesion	normal	Well-defined foveal hypoautofluorescent zone	choroidal hyperreflective cone	-
P2	5	6/10	normal	-	-	-	-	Bilateral depigmented foveolar lesion with well-defined borders	normal	Well-defined foveal hypoautofluorescent zone	Thickening of the *umbo*	-
P3	5	8/10	normal	-	-	-	normal	Bilateral hypopigmented foveolar lesion surrounded by a slightly darker halo	normal	Well-defined foveal hypoautofluorescent zone	choroidal hyperreflective cone	5
P4	9	12/10	normal	normal	normal	normal	normal	Bilateral hypopigmented foveolar lesion measuring half a disc diameter with irregular borders	normal	Well-defined foveal hypoautofluorescent zone	choroidal hyperreflective cone	4

### Patient 1

**Fig. 1:**
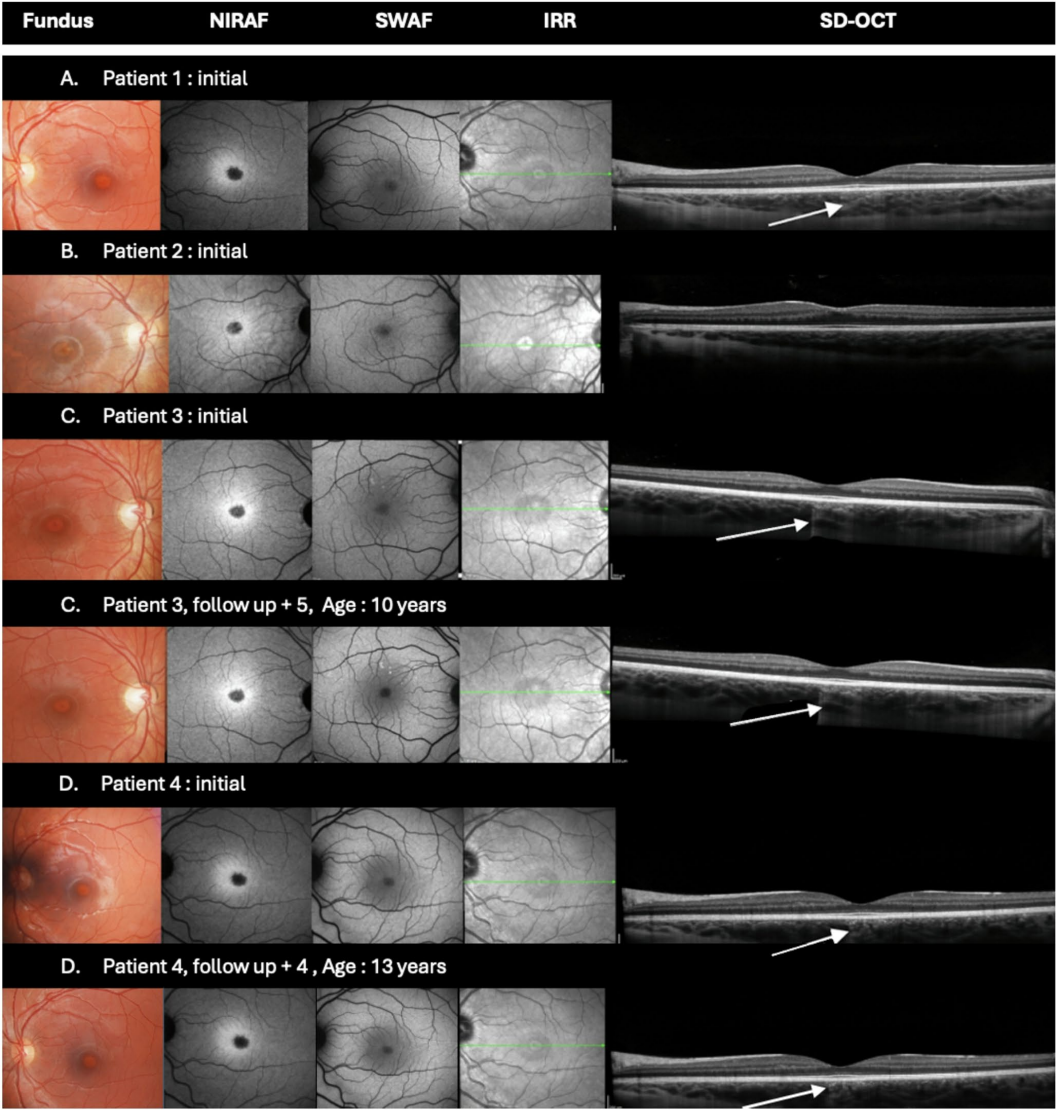
Multimodal imaging data of patients (**A**) Patient 1. Small hypopigmented foveal lesion on fundus examination. Well-defined hypoautofluorescent foveal lesion on near-infrared autofluorescence (NIRAF). Normal short-wavelength autofluorescence (SWAF). Choroidal hyperreflective cone visible on SD-OCT (**B**) Patient 2. Small hypopigmented foveal lesion on fundus examination. Well-defined hypoautofluorescent foveal lesion on NIRAF. Normal SWAF. Thickening of the internal limiting membrane of the umbo on macular OCT (**C**) Patient 3. Hypopigmented foveal lesion approximately half the disc diameter with irregular borders on fundus examination. Well-defined hypoautofluorescent foveal area on NIRAF. Normal SWAF. Choroidal hyperreflective cone adjacent to the foveal lesion on SD-OCT (arrow). Imaging findings are unchanged at 5-years follow-up assessment (age 10 years old) (**D**) Patient 4. Hypopigmented foveal lesion approximately half the disc diameter with irregular borders on fundus examination. Well-defined hypoautofluorescent foveal area on NIRAF. Normal SWAF. Choroidal hyperreflective cone adjacent to the foveal lesion on OCT (arrow). At four-year follow-up (age 13 years old), unchanged imaging findings. Imaging of the patients

A 3-year-old female patient was referred for assessment of a “bull’s-eye” maculopathy. She had no significant personal or family medical history and reported no visual complaints. BCVA was 0.6 OU (*oculus uterque*, each eye). Anterior segment examination was unremarkable. Fundus examination revealed a small yellowish foveolar lesion in both eyes. SWAF was normal; NIRAF imaging found a well-demarcated hypoautofluorescent foveal lesion. Spectral-domain optical coherence tomography (SD-OCT) demonstrated a normal reflective structure of the foveolar retinal layers; however, a cone of choroidal hyperreflectivity corresponding to the foveal lesion was observed (Fig. 1.A).

### Patient 2

A 5-year-old female patient was referred for evaluation of an incidentally discovered maculopathy. She had no medical history apart from a behavioral disorder (ADHD). BCVA was 0.6 OU. Fundus examination revealed a well-demarcated depigmented foveal lesion. Full-field electroretinography (ffERG) was normal (eFig. 5, Supplement). SWAF was normal; NIRAF found a clearly defined hypoautofluorescent foveal lesion. SD-OCT revealed a thickening of the *umbo*. The structure of the other reflective retinal layers was normal (Fig. 1.B).

### Patient 3

A 5-year-old female patient was referred for assessment following the incidental discovery of bilateral yellowish macular spots. She had no significant personal or family medical history and was asymptomatic. BCVA was 0.8 OU. Fundus examination revealed a hypopigmented foveolar lesion surrounded by a slightly darker halo. ffERG was normal. SWAF was normal in the fovea; however, a few unilateral hyperautofluorescent lesions were observed along the superior temporal vascular arcade. NIRAF revealed a well-defined, round hypoautofluorescent foveal lesion. SD-OCT showed a normal reflective structure of the fovea. A cone of choroidal hyperreflectivity corresponding to the foveal lesion was present (Fig. 1.C). The patient was re-evaluated at the age of 10. She remained asymptomatic. BCVA was 1.2, and both structural and functional assessments remained unchanged (Fig. 1.C).

### Patient 4

A 9-year-old female patient was referred for assessment of a bilateral maculopathy incidentally detected by her ophthalmologist. Stargardt disease had initially been suspected. The patient had no relevant medical or surgical history. Her parents had no known ocular conditions other than uncomplicated myopia. The patient was asymptomatic. BCVA was 1.0 OU. Static visual field was normal OU (eFigs. 1 and 2, Supplement). Electrophysiological assessment of macular function, including pERG and mfERG, was strictly normal (eFigs. 3 and 4, Supplement). Fundus examination revealed a hypopigmented foveal lesion with irregular borders, measuring approximately half of a disc diameter. No peripheral retinal or optic disc abnormalities were noted. SWAF was normal; NIRAF showed a well-demarcated hypoautofluorescent foveal area corresponding to the lesion observed on the fundoscopy. Retinal reflective layers appeared normal on SD-OCT. However, a cone of choroidal hyperreflectivity was present in the area corresponding to the foveal lesion (Fig. 1.D).

The patient was re-evaluated four years later, at the age of 13. She remained asymptomatic, with a 1.0 BCVA, and no changes were observed in the imaging or functional assessments (Fig. 1.D and eFig. 2, Supplement).

The parents and siblings of the affected children were also examined. No cases of familial involvement were identified.

No pathogenic variants were identified in P2 and P3, tested on a large IRD panel.

## Discussion

We reported four pediatric cases of benign foveal depigmentation. As for the previously reported cases by Boulanger et al. [1], Parodie et al. [2], Fantaguzzi et al. [3], and Arend et al. [4], our patients had no functional visual impairment associated with the macular lesions. All patients were asymptomatic, and the lesions were discovered incidentally during routine eye examinations. Additionally, there were no relevant personal or family ocular condition histories.

All four children in our series were females. Previously reported adult cases of macular depigmentation in the literature [1, 3] were exclusively women aged between 23 and 65 years. Arend [4] was the only author to describe this condition in two pediatric patients, one of whom was male. BFD thus appears to predominantly affect females, both in children and adults.

In our series, the maculopathy was bilateral and symmetrical in all cases. However, Boulanger et al. demonstrated that the condition can also present unilaterally [1].The retinal lesions were characterized by foveal hypoautofluorescence on near-infrared autofluorescence imaging. In contrast to previously reported cases [1], short-wavelength autofluorescence images were normal in all of our patients. The abnormalities were only visible on NIRAF, suggesting a localized deficiency of foveal melanin [7].

On OCT, a cone of choroidal hyperreflectivity corresponding to the foveal lesion was observed in 3 out of 4 patients; however, this feature was absent in Patient 2. This may be explained by a more limited area of macular depigmentation in this case. Additionally, an umbo thickening and internal limiting membrane (ILM) duplication were noted in Patient 2, a finding not previously reported in the literature. One patient (Patient 3) also presented few unilateral hyperautofluorescent lesions along the superior temporal vascular arcade on SWAF imaging. These lesions have not been described in the literature and were not found in our other cases. These findings are likely unrelated to BFD.

Visual field testing could not be performed in all patients due to their young age. When possible, it was normal as well. Electrophysiological assessments—including full-field, pattern and multifocal ERG—were strictly normal, ruling out any generalized or localized retinal dystrophy/dysfunction associated with the maculopathy. This finding is entirely consistent with previously published data [1, 4].

The very young age of Patient 1 at the time of lesion discovery suggests an early, likely congenital onset of the condition. Foveal lesions remained stable—with no change in shape or size—throughout the follow-up period in Patients 3 and 4 (five and four years of follow-up, respectively). Arend et al. [3] reported patients followed from five to ten years: a more pronounced depigmentation in older individuals compared to younger patients was noted, suggesting a progressive depigmentation over time, although the visual function remained normal.

The origin of BFD remains poorly understood. According to Parodi [2], there may be localized alterations restricted to the retinal pigment epithelium (RPE) layer, which could be detectable on OCT and suggest a defect in the local production or distribution of melanin within RPE cells. It is possible that a defect in melanin production or distribution exists at the level of the RPE cells without any impact on the thickness or continuity of the retinal reflective layers on OCT. Furthermore, Fantaguzzi et al. [3] proposed a dysregulation of melanin production in the choroid that may coexist with melanin abnormalities in RPE cells. This could explain the cone of choroidal hyperreflectivity underlying the hypopigmented foveal lesion on OCT. Choroidal melanin abnormalities may also affect choroidal reflectivity on OCT without necessarily being associated with any significant retinal alterations.

The main differential diagnosis is an early-stage Stargardt disease. This disorder was initially suspected in Patients 1 and 4 but was ultimately ruled out, particularly due to the absence of visual impairment or symptoms, the presence of typical features of BFD on multimodal imaging, and, most importantly, the lack of progression [8]. Other depigmented lesions may present with a similar fundoscopic appearance, such as fenestrated sheen macular dystrophy [9, 10]. Its early fundus features include a shimmering yellowish foveolar lesion with smaller reddish lacunae, progressing to marked perifoveal atrophy. However, this foveal lesion differs from those observed in our patients in terms of family history, the presence of RPE disruption, and peripheral retinal pigment epithelial changes — none of which were present in our cases. Benign concentric annular macular dystrophy (BCAMD) is another differential diagnosis, but it differs from our cases by its autosomal dominant inheritance pattern and the presence of photoreceptor degeneration, which is clearly visible on OCT [11].

## Conclusion

Benign foveal depigmentation is a rare retinal anomaly, insufficiently described in the literature and little known, yet carrying a favorable prognosis. We reported four pediatric patients with BFD. The diagnosis remains one of exclusion. Multimodal imaging, particularly the round hypoautofluorescent aspect on near-infrared autofluorescence associated with a posterior shadow cone on SD-OCT, is specific for BFD. The absence of functional impairment and long-term stability confirms the benign nature of this peculiar foveal anomaly.

## Supplementary Information

Below is the link to the electronic supplementary material.

Supplementary File 1 (DOCX 9.70 MB)

## Data Availability

No datasets were generated or analysed during the current study.
