# Supplementary material for: Benign foveal retinal pigment epithelium hypopigmentation without functional loss : pediatric case series
Source: Graefes Arch Clin Exp Ophthalmol. 2026 Jan 10;264(5):1429–34. doi: 10.1007/s00417-025-07074-3 (PMC13091843; doi:10.1007/s00417-025-07074-3)
Supplement: Supplementary file 1 — (DOCX 9.70 MB) [file 417_2025_7074_MOESM1_ESM.docx]

Supplement

INDEX

eFigure 1 1

eFigure 2 2

eFigure 3 2

eFigure 4 3

eFigure 5 4

eFigure 6 5

| OD | OS |
| --- | --- |
| 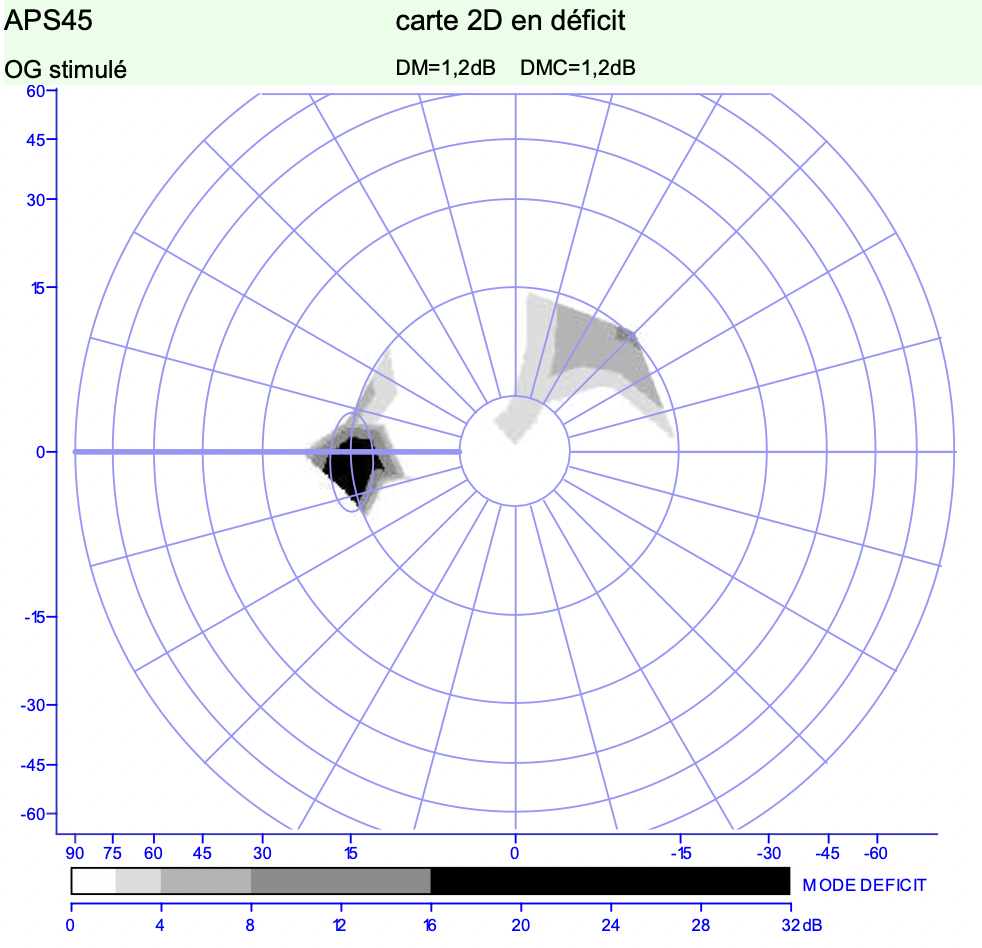 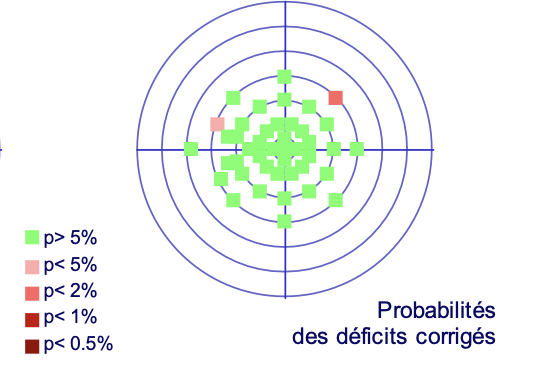 | 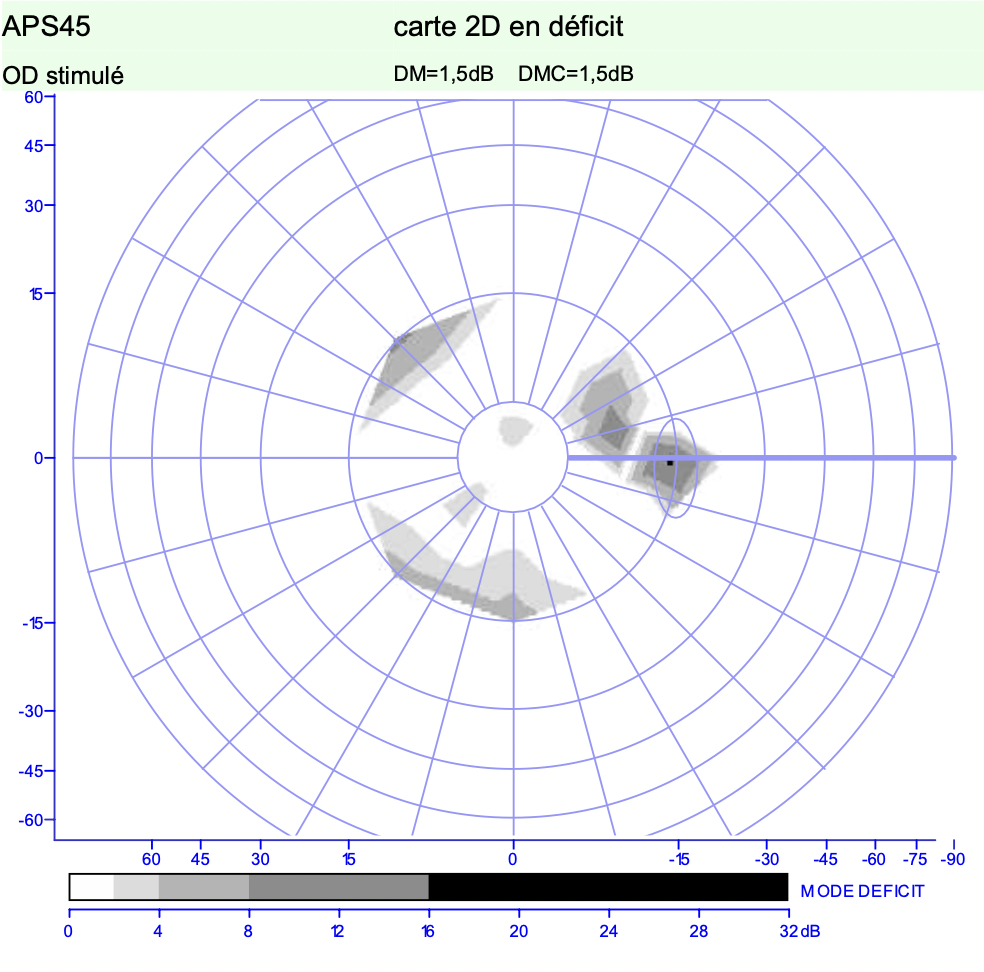 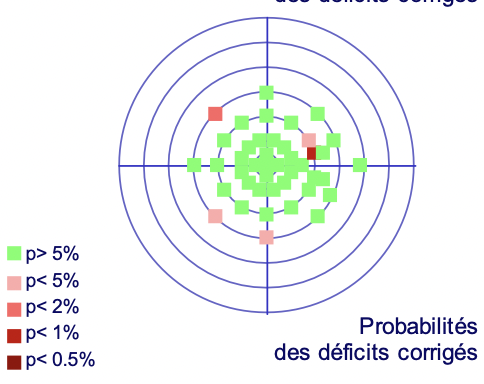 |

**eFigure 1. Visual field of Patient 4 at initial assessment.** Age: 9 years old

| OD | OS |
| --- | --- |
| 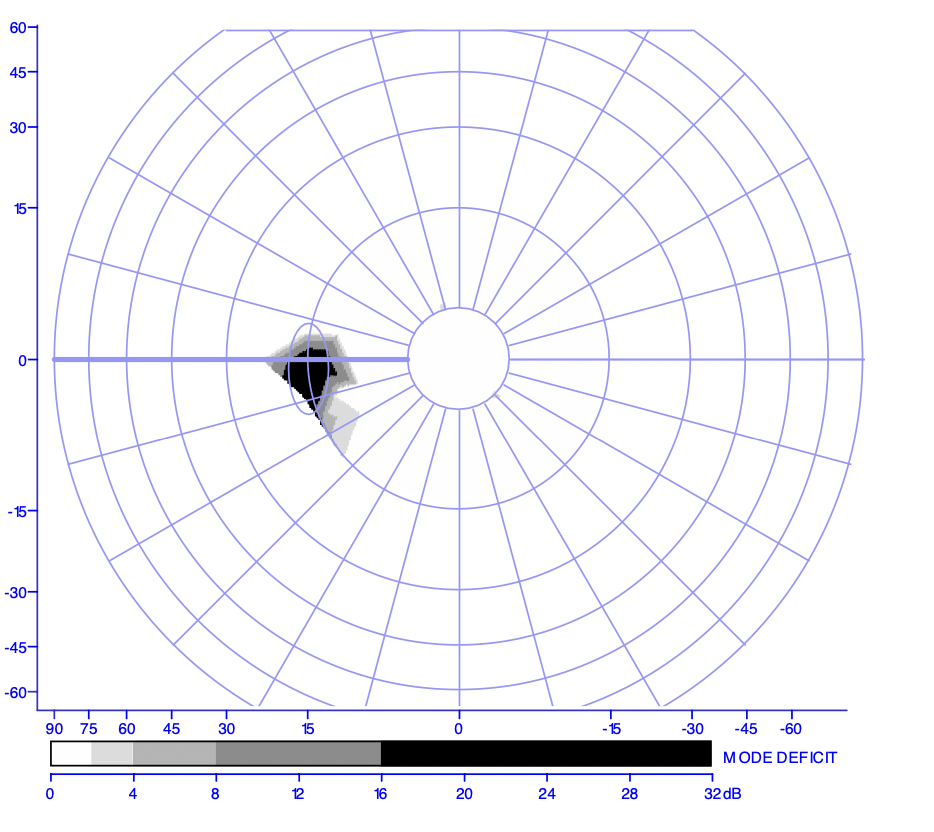 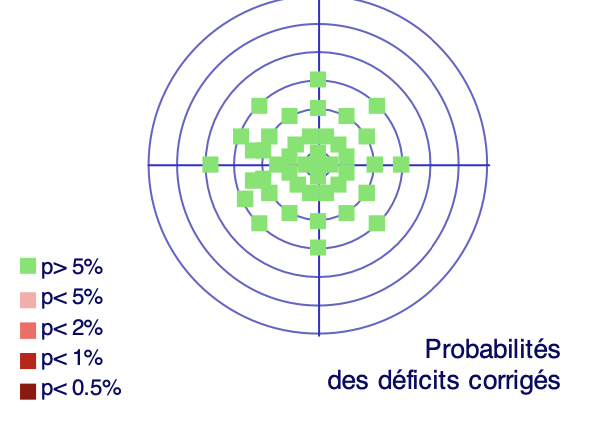 | 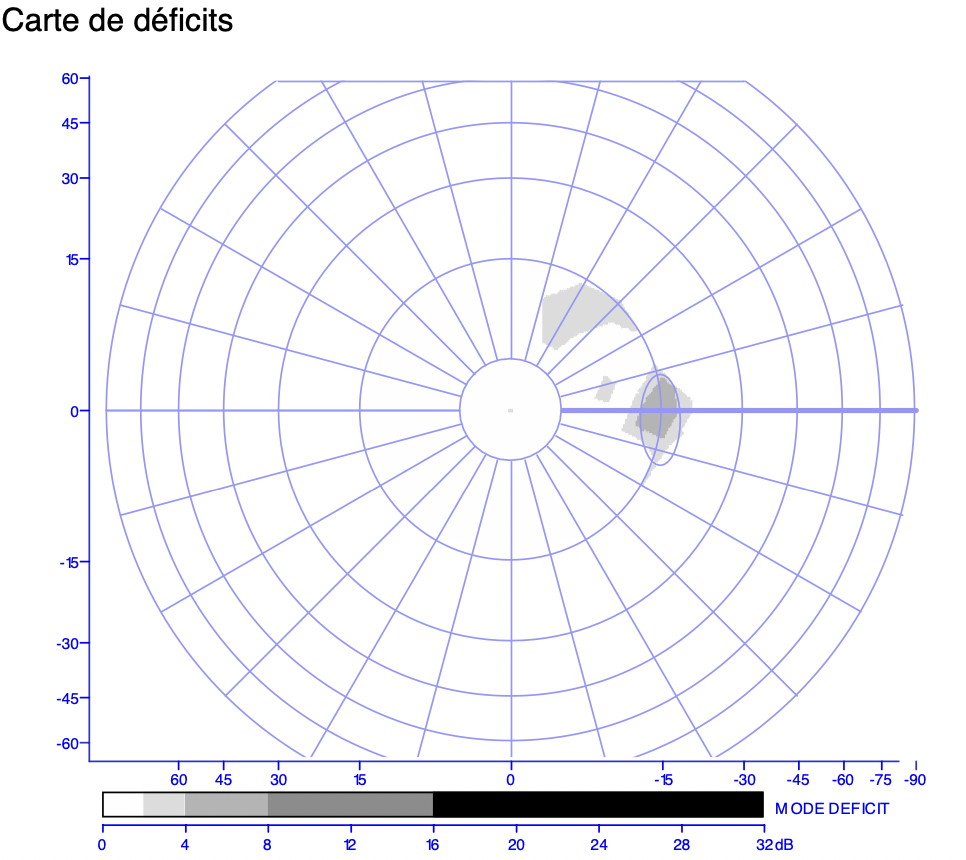 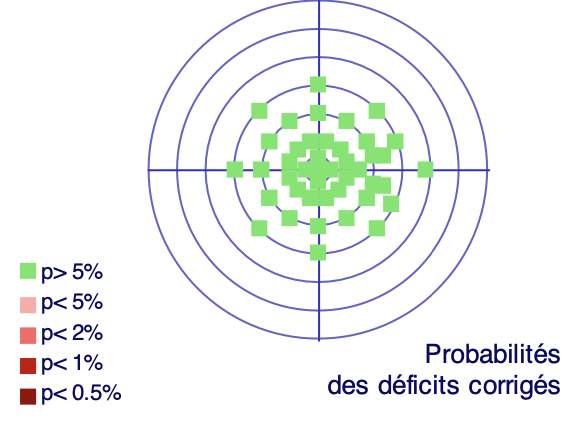 |

**eFigure 2.** **Visual field of Patient 4 at follow-up.** Age : 13 years old.

**eFigure 1 and 2** : Static visual field within the central 10 degrees in P4 at 9 years of age (Figure 1) and 13 years of age (Figure 2), showing a subnormal visual field (compliance errors) at age 9 and a normal central visual field at age 13.

| OD |
| --- |
| 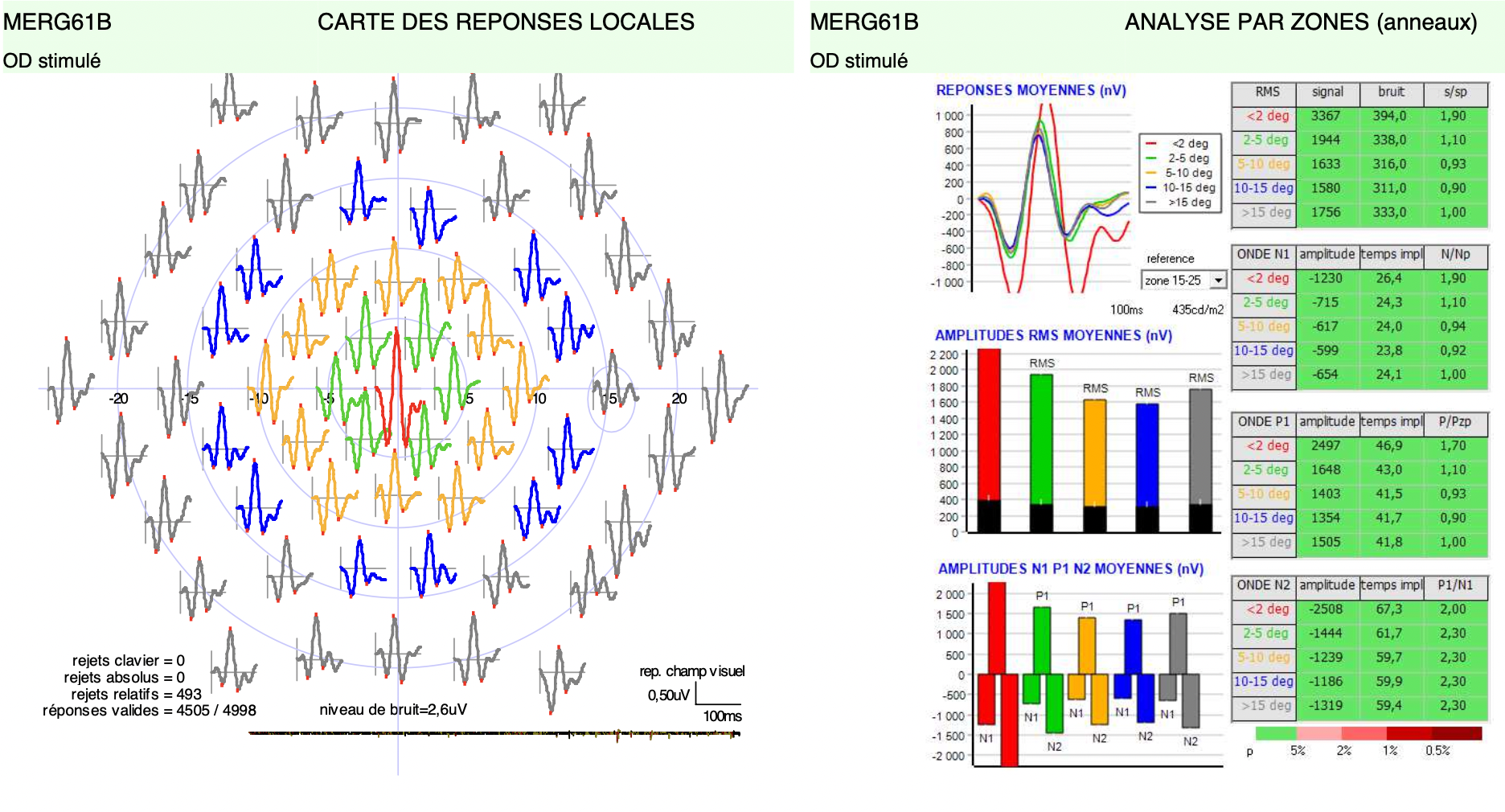 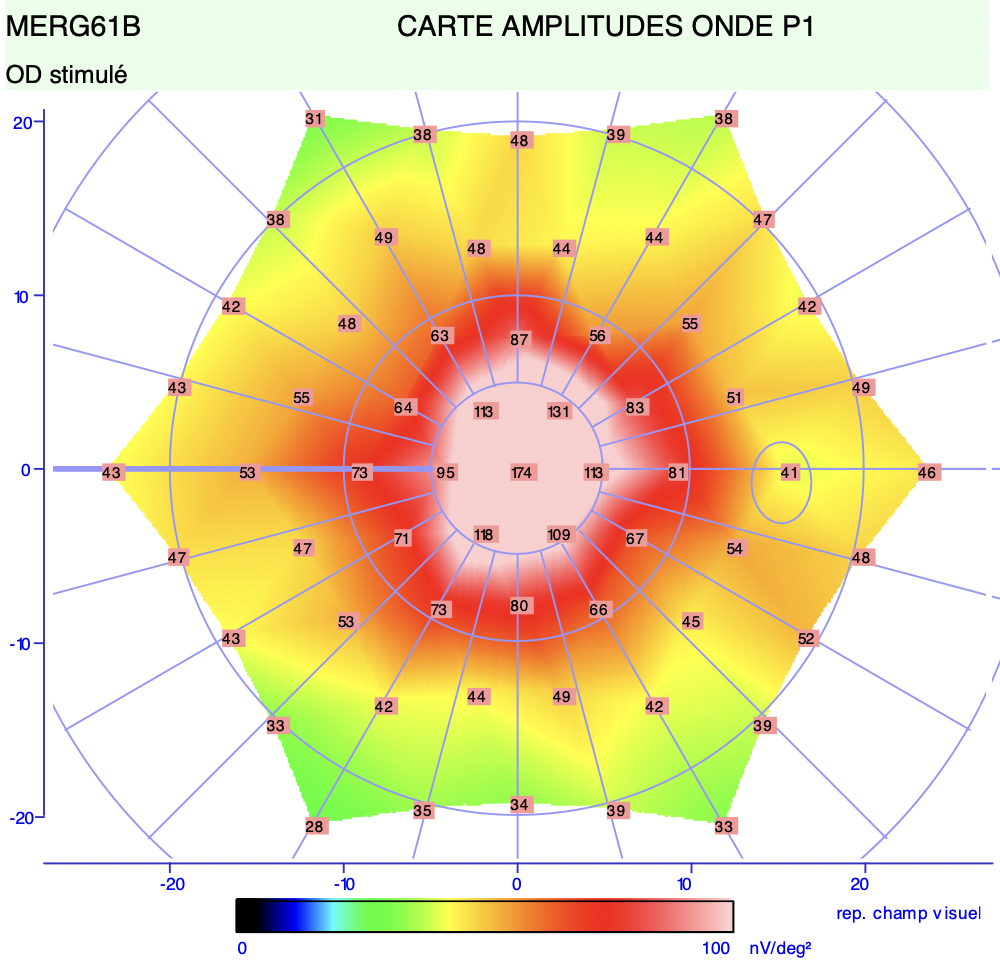 |
| OS |
| 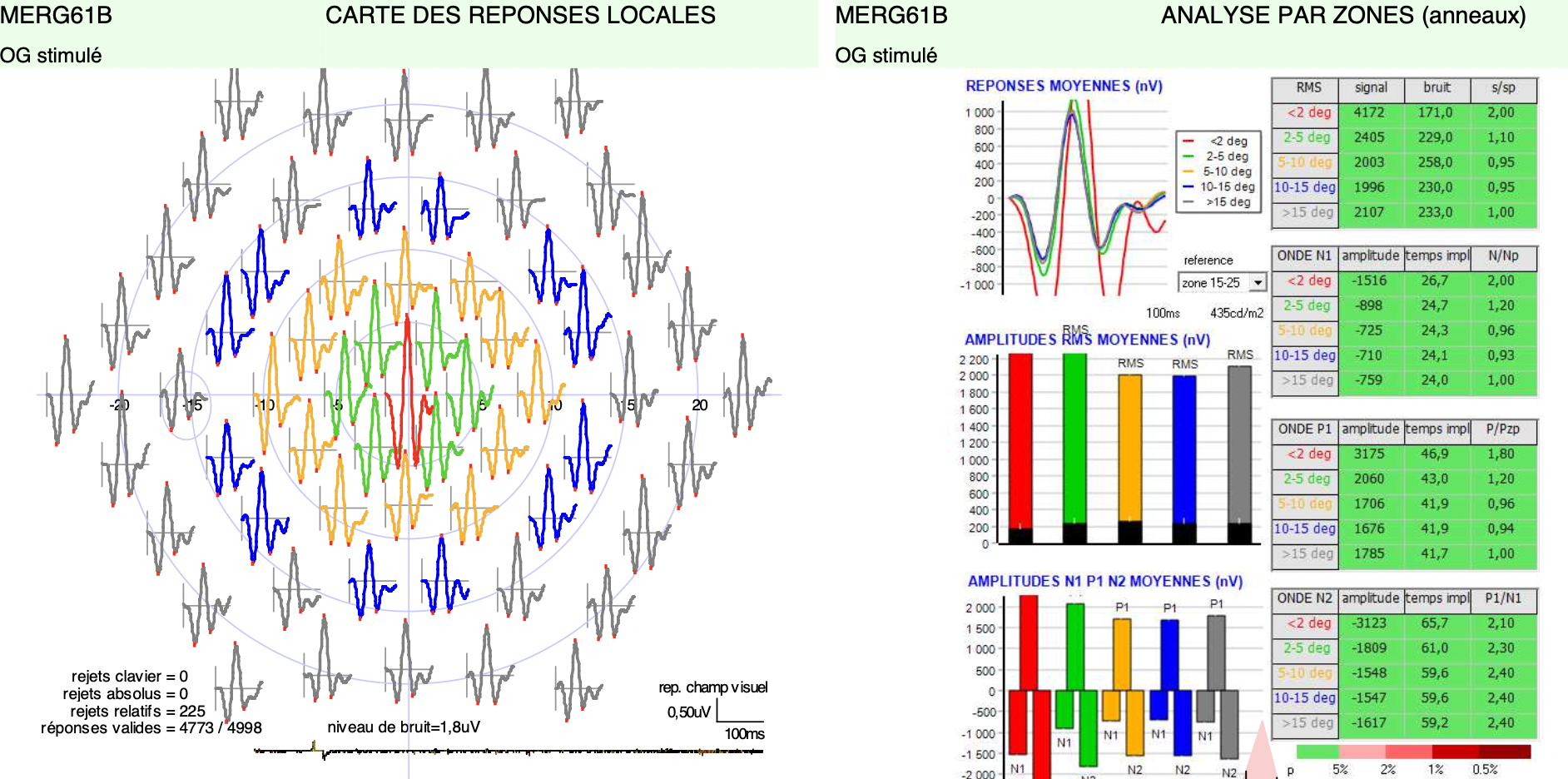 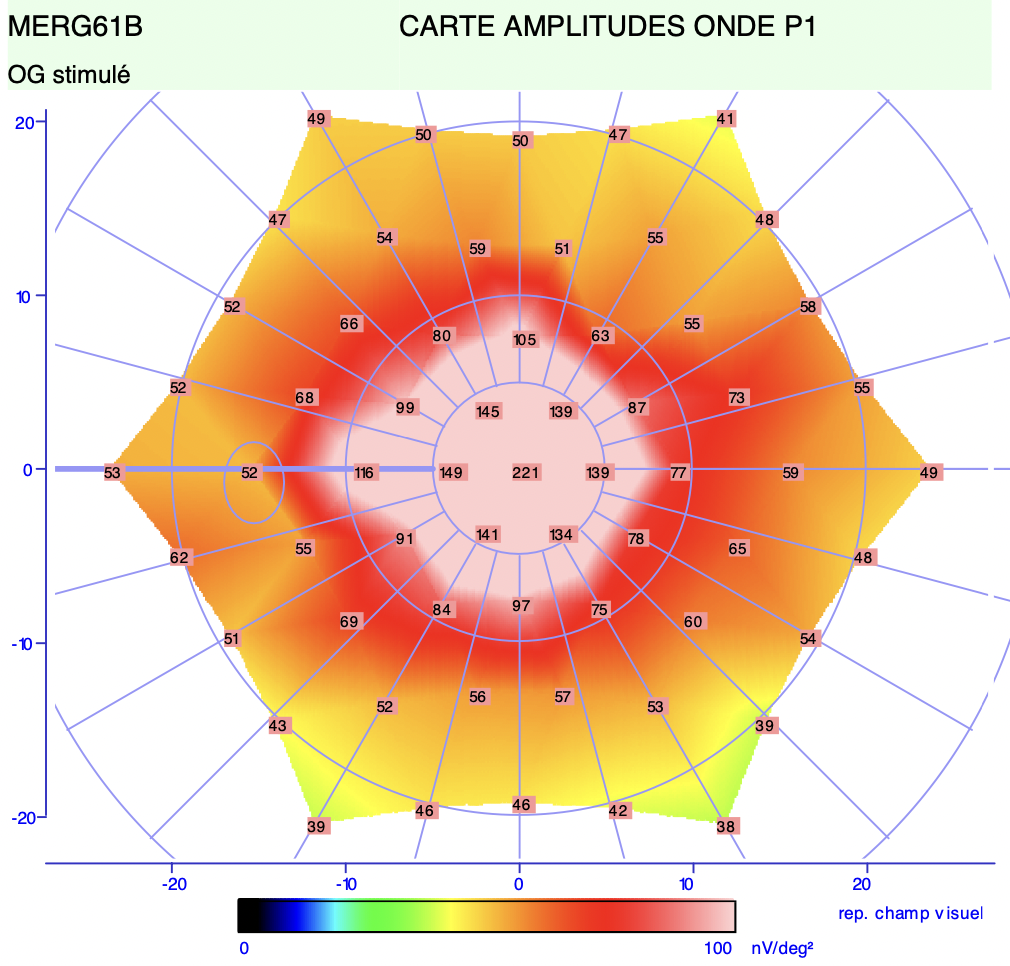 |

**eFigure 3 : Multifocal ERG of P4**. Normal response with a well-defined foveal peak, indicating proper function of ON and OFF bipolar cells connected to cone photoreceptors.


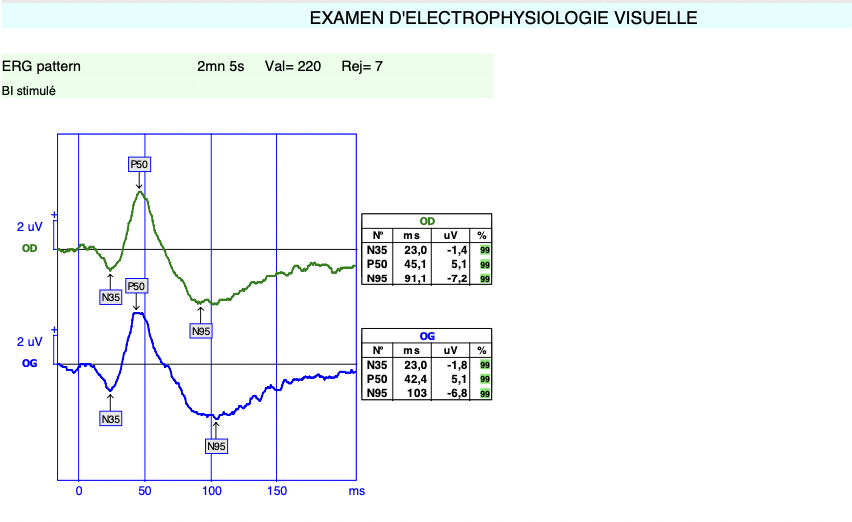
 **eFigure 4. Pattern ERG of Patient 4** . Normal appearance of responses, indicating proper function of macular cones and bipolar cells (P50 wave), as well as ganglion cells (N95 wave).

| Full-field ERG of P3 at age 5 | Full-fiel ERG of a healthy pediatric subject |
| --- | --- |
| 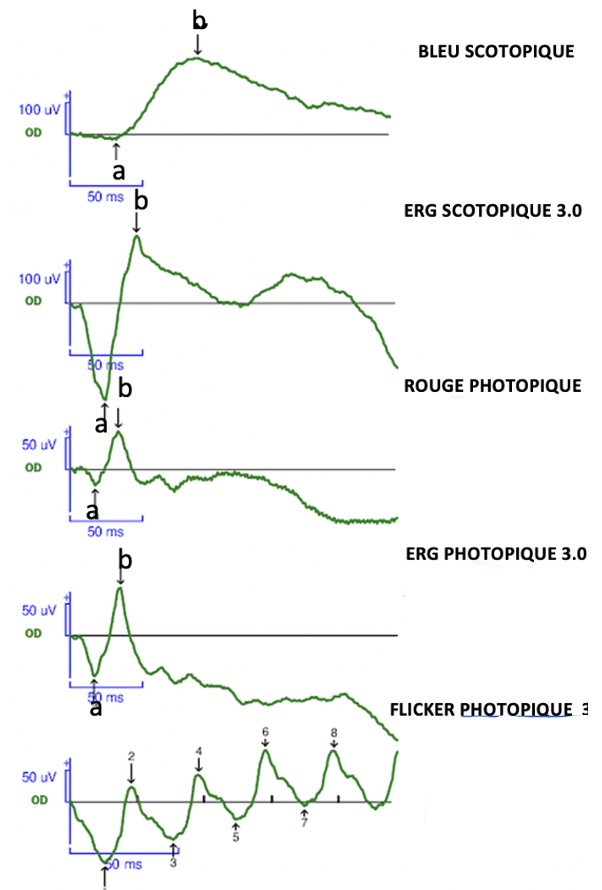 | 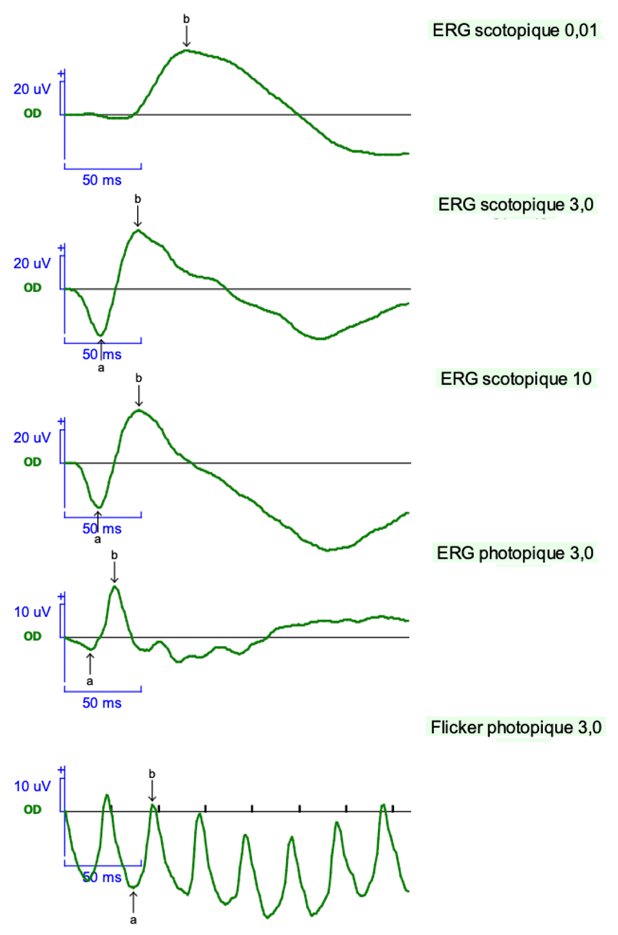 |


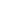

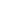

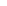

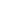

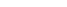

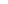

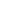


**eFigure 5. ISCEV-standard full-field ERG of Patient 3. Age: 5 years old. Adhesive skin electrodes.** ERG is normal for all stimulations.


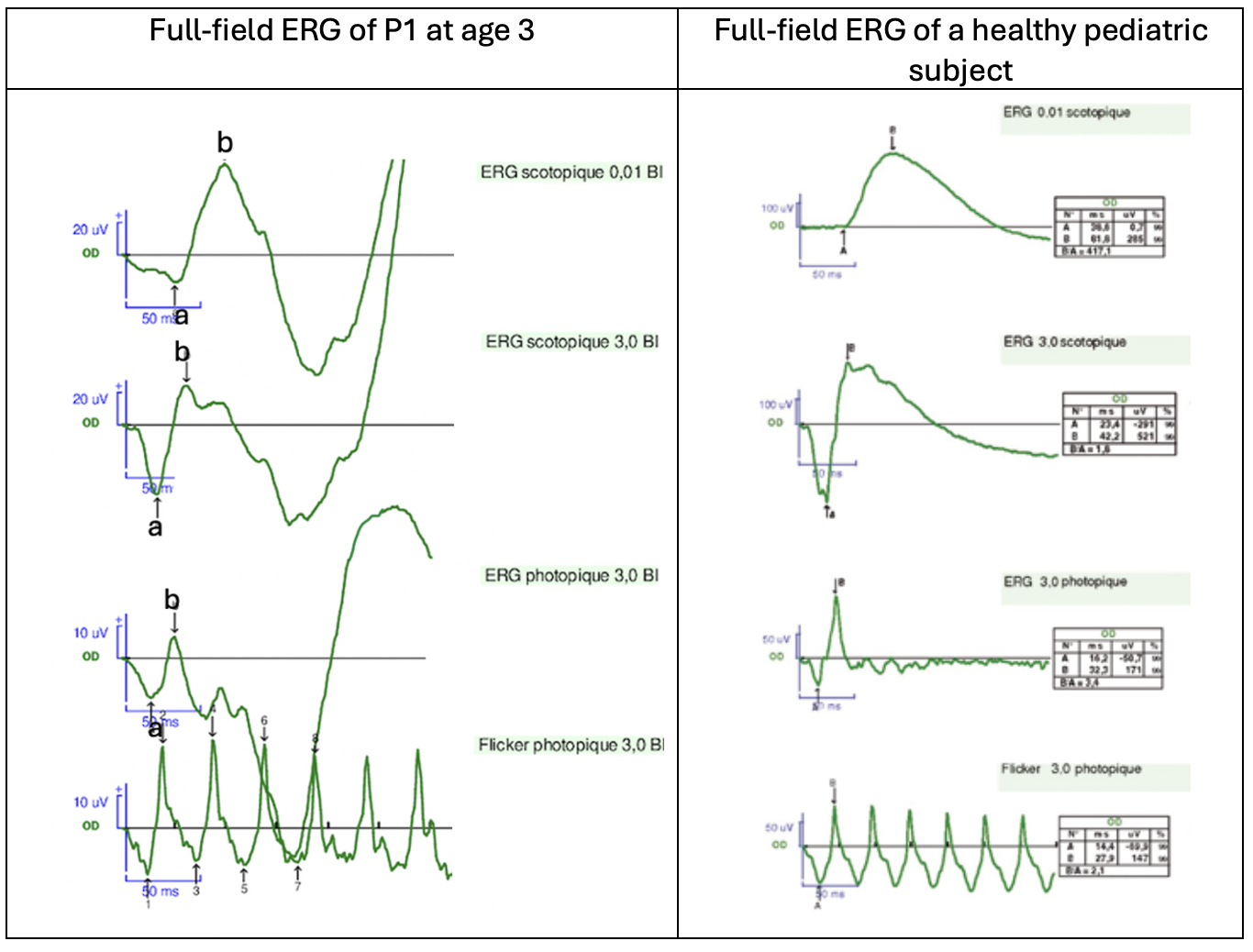


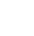

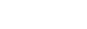

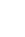


**eFigure 6 : Full-field ERG of P1 at age 3. Normal, ruling out an associated retinal dystrophy.**

Benign foveal retinal pigment epithelium hypopigmentation without functional loss: pediatric case series.

Emilie Boulert^1^, Isabelle Drumare^1^, Claire-Marie Dhaenens^2 ,3^ , Sabine Defoort-Dhellemmes^1^, Vasily Smirnov^1,2^

^1^CHU Lille, Department of Vision Exploration and Neuro-Ophthalmology, Hôpital Salengro, 59037 Lille, France

^2^Univ. Lille, Inserm, CHU Lille, U1172-LilNCog-Lille Neuroscience & Cognition, 59045 Lille, France

^3^Univ. Lille, CHU Lille, Department of Toxicology and Genetic Diseases, 59037 Lille, France.

**Corresponding author:**
Vasily Smirnov
e-mail : vasily.smirnov@chu-lille.fr
Postal address: CHU Lille, Department of Vision Exploration and Neuro-Ophthalmology, Hôpital Salengro, 59037 Lille, France
Telephone: [+33 6 03 37 79 82
